# A Chronoamperometric Screen Printed Carbon Biosensor Based on Alkaline Phosphatase Inhibition for W(VI) Determination in Water, Using 2-Phospho-l-Ascorbic Acid Trisodium Salt as a Substrate

**DOI:** 10.3390/s150202232

**Published:** 2015-01-22

**Authors:** Ana Lorena Alvarado-Gámez, María Asunción Alonso-Lomillo, Olga Domínguez-Renedo, María Julia Arcos-Martínez

**Affiliations:** 1 School of Chemistry & CELEQ, University of Costa Rica, San Pedro de Montes de Oca, 11500-2060 San José, Costa Rica; 2 Department of Chemistry, Faculty of Sciences, University of Burgos, Plaza Misael Bañuelos s/n, 09001 Burgos, Spain; E-Mails: malomillo@ubu.es (M.A.A.-L.); olgado@ubu.es (O.D.-R.); jarcos@ubu.es (M.J.A.-M.)

**Keywords:** alkaline phosphatase, chronoamperometry, 2-phospho-l-ascorbic acid trisodium salt, screen printed carbon electrode, tungsten

## Abstract

This paper presents a chronoamperometric method to determine tungsten in water using screen-printed carbon electrodes modified with gold nanoparticles and cross linked alkaline phosphatase immobilized in the working electrode. Enzymatic activity over 2-phospho-l-ascorbic acid trisodium salt, used as substrate, was affected by tungsten ions, which resulted in a decrease of chronoamperometric current, when a potential of 200 mV was applied on 10 mM of substrate in a Tris HCl buffer pH 8.00 and 0.36 M of KCl. Calibration curves for the electrochemical method validation, give a reproducibility of 5.2% (*n* = 3), a repeatability of 9.4% (*n* = 3) and a detection limit of 0.29 ± 0.01 μM. Enriched tap water, purified laboratory water and bottled drinking water, with a certified tungsten reference solution traceable to NIST, gave a recovery of 97.1%, 99.1% and 99.1% respectively (*n* = 4 in each case) and a dynamic range from 0.6 to 30 μM. This study was performed by means of a Lineweaver–Burk plot, showing a mixed kinetic inhibition.

## 1. Introduction

Tungsten is a metal which occurs naturally in soils and sediments, usually in small concentrations ranging between 0.2 and 2.4 mg·kg^−1^ in the lithosphere [1–3]. This metal is also present in oceanic waters in trace amounts, for instance its contents for the Northern Atlantic and Pacific Oceans reported in the literature are 100 ng·L^−1^ and 8 ng·L^−1^ respectively [1]. In aqueous media, WO_4_^2−^ is the dominant species, occurring as monomer in a pH range of 6.9 to 9.3, while polytungstate species form at lower pH and higher W concentration. Ions such as NH_4_^+^ shift the pH at which polycondensation occurs, thus polytungstate species in ammoniacal solution are stable at pH values where normally only monotungstate ions would exist. On the other hand, W has been found with a concentration between 0.27 and 742 μM in groundwater in Carson Desert (Nevada, USA) [4].

Natural processes for tungsten isolation include weathering of W-rich rocks and soils, dissolutions, hydrothermal and volcanic activity, atmospheric precipitation (wet and dry) and excretion of metabolites. Tungsten occurs in the oxidation states III, IV, V and VI, however, oxidation state VI represents the most stable of tungsten species. It has tensile strength at high temperature, a density of 19.1 g·cm^−1^ and the highest melting/boiling points among elements. These properties make tungsten suitable for a wide variety of uses. Tungsten-based products have been in use in a wide range of applications, stretching from daily household supplies to highly specialized components of modern science and technology [1].

For a long time, tungsten was considered an insoluble metal that did not exhibit serious toxicological or environmental effects, and it was placed among less toxic elements. Nevertheless tungsten effects on environmental systems have not been investigated extensively in regards to its ecotoxicological effects, so published data are fragmentary. In fact, anthropogenic sources include a variety of industrial, commercial and military activities along with non-sustainable disposal practices of municipal, agricultural and industrial wastes. However, in spite of its extensive uses, biological and biochemical effects of tungsten and tungsten compounds are not well known [1,5].

For instance, it has been reported recently that tungsten as a trace element is toxic to people and animals, such as 5 μg·kg^−1^ of tungsten led to the death of animal embryos [2,6,7]. Other studies have shown that dissolution of metallic tungsten particles may cause adverse environmental effects such as soil acidification as well as direct and indirect toxic effects in plants, soil microorganisms and invertebrates. Therefore, it has been found necessary to re-evaluate the environmental regulations of tungsten, to confirm the “nontoxic” and “environmentally inert” of the metal [6,8].

There are many techniques to analyse tungsten in environmental samples; mostly spectrophotometric methods such as Atomic Absorption and ICP-OES, as well as voltammetric and polarographic methods have been used [2,3,9–11]. The application of enzyme biosensors for determining toxic compounds is a dynamic promising research trend, because the associated analytical systems are simple, rapid and selective for amperometric, potentiometric or conductimetric techniques. They function by combining an electrochemical process with the activity of an immobilized enzyme. The selected enzyme must provide selectivity through its biological affinity for a particular substrate, usually of biological origin. These type of biosensors constitute one of the most widespread, numerous and successfully commercialized devices in biomolecular electronics since their inception, which implied the development of a novel field in analytical biotechnology [12,13].

The enzyme immobilization on the electrode surface is a critical step that defines an enzyme electrode's effectiveness. Different chemical, biochemical and physical factors such as the chemical cross linking with glutaraldehyde, other functional antigen–antibody agent interactions, as well as the magnetic interactions, entrapment or encapsulation within polymers and the formation of paste materials, are generally used to immobilize the enzyme on the electrode surface [9,13].

Alkaline phosphatase has been used to analyse some heavy metals, such as vanadium and molybdenum through these techniques. Metals such as vanadium and tungsten exert inhibitory effects on the enzyme alkaline phosphatase (ALP). For instance, in 1974, Van Etten [14] demonstrated the influence of vanadate, molybdate and tungstate on phosphohydrolases such as acid phosphatases which are relatively nonspecific enzymes that catalyze the hydrolysis of several alkyl and aryl phosphate esters at pH values between 4 and 6. Our research group recently reported promising results using two different biosensors with this enzyme for vanadium detection at trace levels in water [15,16], studying modification of electrode surfaces with gold nanoparticles in order to provide a micro-environment similar to native systems of redox proteins and allowing these molecules more freedom in orientation, thereby reducing the insulating effect of the protein shell towards direct electron transfer through gold nanocrystal conducting tunnels [17–19].

Thereby, we report in this paper the development of a new methodology to determine tungsten in water at low μmol·L^−1^ concentrations using a biosensor with a disposable screen printed carbon electrode (SPCE) modified with gold nanoparticles, using the enzyme alkaline phosphatase immobilized over the working electrode and 2-phospho-l-ascorbic acid trisodium salt as substrate.

## 2. Experimental Section

### 2.1. Chemical Reagents

Several inks were used in the fabrication of the screen printed carbon electrodes (SPCEs), namely Electrodag PF-407 A (carbon ink), Electrodag 6037 SS (silver/silver chloride ink) and Electrodag 452 SS (dielectric ink), all supplied by Acheson Colloiden (Scheemda, The Netherlands). Analytical grade chemicals with no further purification were used. All solutions were prepared in ultrapure water, conductivity of 0.05 μS/cm (Gen-Pure TKA, Niederelbert, Germany).

Hydrogen tetrachloroaurate (III) trihydrate (HAuCl_4_), alkaline phosphatase (ALP), bovine serum albumine (BSA) and glutaraldehyde (GA) were obtained from Sigma Chemical Co. (St. Louis, MO, USA).

2-Phospho-l-ascorbic acid trisodium salt was acquired from Sigma Aldrich (Steinheim, Germany). Ammonium tungstate CertiPUR traceable to SRM from NIST (Merck, Darmstadt, Germany) was used as stock solution of tungsten. Tungsten metal 99.99% purity, 2% v/v HNO_3_ certified value 1000 ± 4 mg·L^−1^ (CRM traceable to NIST confirmed against SRM 3163, High-Purity Standards, Charleston, SC, USA) was used to spike samples for recovery. 28 mM Tris (hydroxymethyl) aminomethane (Aldrich Chemical Co., Buchs, Switzerland) buffer was used together with 19 mM of MgCl_2_ (Merck) and 0.36 M total Cl^−^ (Merck) as supporting electrolyte. HCl 0.1 mM (Merck) was used to adjust the pH value.

### 2.2. Apparatus

Screen Printed Carbon Electrodes (SPCEs) were produced on a DEK 248 printing machine (DEK, Weymouth, UK) using polyester screens with appropriate stencil designs. Electrochemical measurements were made with an Autolab 128N electrochemical system with GPES software (Echo Chemie, Utrecht, The Netherlands). The pH measurements were performed using a Mettler-Toledo pHmeter S47-K (Columbus, OH, USA).

### 2.3. Software

Data analysis was processed with a Statgraphics Plus (StatPoint Technologies, Inc. Warrenton, VA, USA, 1994–1999) software package for the experimental design process.

### 2.4. Manufacturing of Screen Printed Carbon Electrodes

Home-made SPCEs were used in the determination of tungsten. For the construction of the SPCEs, successive layers of different inks were printed onto a polyester strip substrate following the printing procedure described in previous works [19].

### 2.5. Preparation of Modified SCPEs Biosensor

The working electrode was electrochemically modified by gold nanoparticles (AuNPs), using a 0.1 mM solution of HAuCl_4_ in 0.5 mM H_2_SO_4_. The deposition was performed by applying a potential of +0.18 V (*vs*. Ag/AgCl SPE) during 15 s under stirring conditions [17,19–21]. The biologically sensitive layer of the biosensor was formed by cross-linking the enzyme alkaline phosphatase on the surface of the AuNPs-modified SPCEs, by dropping 10 μL of a 2:1:2 mixture of 0.6% of enzyme solution, 1.75% (w/v) of BSA solution and 2.5% (w/v) of GA solution onto the surface of a screen-printed working electrode. Volume and concentration of ALP, GA and BSA were optimized to obtain the maximum analytical response for the inhibition of the enzyme with W(VI) [15,22]. Finally, the mixture was left to react at 4 °C during one hour and the ALP-AuNPs-SPCEs were stored at 4 °C. Under these storage conditions the developed biosensor showed good stability for at least one week.

### 2.6. Optimization of Experimental Conditions

In order to obtain a sensitive analytical signal, a 2^3^ central composite design was carried out considering three important factors: pH, Cl^−^ and substrate concentrations [23]. A group of 17 experiments were performed at different pH, Cl^−^ and substrate concentrations according to the design, and the best conditions obtained from an estimated surface. Another experiment was carried out to optimize the working potential. Considering that higher potentials are not selective and lead to more oxidized species, we tried to apply a potential as low as possible, varying it from 1.0 V to 0.1 V.

### 2.7. Tungsten Chronoamperometric Determination Procedure

The ALP/SPCEs biosensors were placed in the electrochemical cell containing 5 mL of Tris-HCl buffer solution, pH 8. An adequate potential was applied and, once a steady-state current was set, a defined amount of 2-phospho-l-ascorbic acid trisodium salt 10 mM stock solutions was added to the measuring cell. A large anodic current was observed due to the addition of 2-phospho-l-ascorbic acid trisodium salt. Then, once a plateau corresponding to the steady-state response was reached again, 50 μL aliquots of the tungsten stock solution were added consecutively. The addition of each aliquot resulted in a current decrease proportional to the amount of the metal added. Enzyme electrodes were conditioned in a buffer solution for 5 min between each calibration setting.

### 2.8. Validation

Several calibration curves with the same and different electrodes were used to evaluate the figures of merit. To establish the accuracy, reliability, and reproducibility of the collected data, all tests were recorded in triplicate and only average values are reported. Blank tests were run in parallel. All the lab ware used in the study was previously soaked in Alconox, rinsed with distilled water, and finally with ultrapure water from TKA and allowed to dry at room temperature.

## 3. Results and Discussion

To develop the alkaline phosphatase biosensor, initially two substrates were used, p-nitrophenylphosphate, and 2-phospho-l-ascorbic acid trisodium salt, because in previous papers we reported its use to determine V(V) and As(V) [15,24]. Based on different experiments results, we decided to work with 2-phospho-l-ascorbic acid trisodium salt, which had been tested only with acid phosphatase, but not with the alkaline enzyme. To the best of authors' knowledge, the determination of tungsten using biosensors based on the inhibitory effect of this metal over ALP have not been described up to now. The alkaline phosphatase biosensor is based on the following reaction:

$$\begin{array}{c} \text{alkaline phosphatase}\\ R-PO_{3}^{2-}+H_{2}O↔R-H+HPO_{4}^{2-} \end{array}$$

where R-PO_3_^2−^ is the organic phosphate substrate and HPO_4_^2−^ the monohydrophosphate. When *p*-nitrophenyl phosphate is used as substrate, the product RH is *p*-nitrophenol and when 2-phospho-l-ascorbic acid is used as substrate, the product R-H is l-ascorbic acid. Therefore, in the presence of alkaline phosphatase, the reaction induces to a change in the pH and in conductivity [25].

It is well-known that chronoamperometric measurements are influenced by different factors such as pH of the medium, the applied potential, the substrate concentration, and also ionic strength among others, depending on the studied system. Based on the optimization process to obtain the best current signals, the optimized parameters are summarized in Table 1, which were used in tungsten determination: supporting electrolyte pH 8.00, working potential of +0.20 V *vs*. Ag/AgCl SPE, a substrate concentration of 0.32 mM and Cl^−^ concentration of 0.36 M. Easily quantifiable chronoamperometric signals are registered under these optimized conditions for tungsten.

Table 2 show higher calibration curve slopes at lower applied potentials, thus, 0.20 V was the best potential for our experiments. To analyse the signal with the immobilized enzyme, control experiments were carried out under the optimum conditions using bare SPCEs and AuNPs-SPCEs but without the enzyme. No analytical signal was obtained; hence the inhibition response registered after the addition of the substrate is only related to tungsten concentration. Consequently, tungsten can be determined by its inhibitory effect on the response of ALP to 2-phospho-l-ascorbic acid trisodium salt, by calibration curves as it is shown in Table 2.

**Figure 1.**
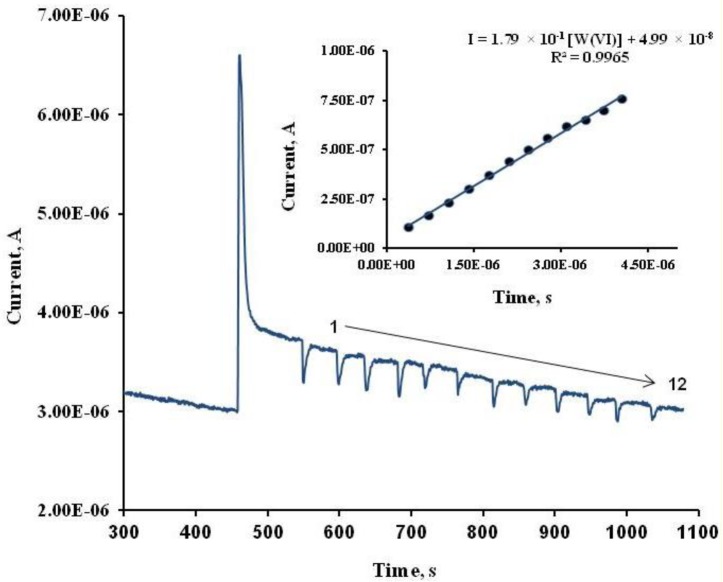
Chronoamperogram registered using an ALP-based biosensor under the optimum conditions (applied potential, +0.20 V *vs*. Ag/AgCl SPE; supporting electrolyte pH 8.00 (Tris HCl buffer, 0.36 M total Cl^−^) and 2-phospho-l-ascorbic acid trisodium salt 0.32 mM, in the W(VI) concentration range from 3.0 μM to 30.0 μM. Inset figure, a calibration curve for twelve aliquot W(VI) additions under optimum conditions.

Figure 1 represents a chronoamperogram obtained for alkaline phosphatase biosensor, a base line represents the current of the buffer signal, the first current step is due to substrate addition, and the rest, 1 to 12, resembles consecutive additions of aliquots of W(VI) standard. The inset figure is the calibration curve of this chronoamperogram, experimental conditions are indicated in the graph.

### 3.1. Inhibitory Effect of W(VI) Over the ALP Enzyme

The inhibitory effect of W in the enzymatic activity, when using 2-phospho-l-ascorbic acid trisodium salt as substrate, was studied by means of kinetic parameters of the Lineweaver-Burk plot. The y-intercept of this graph is equivalent to the inverse of *V_max_*, the x-intercept of the graph represents −*1/K_m_* (*K_m_* is the Michaelis–Menten constant and *V_max_* is the maximum reaction velocity). It also gives a quick, visual impression of the different forms of enzyme inhibition, both in absence and presence of tungsten. This study was performed under different ionic strength, according to values of *Km* and slopes, suggesting a mixed inhibition [26,27]. In fact, the inhibitory effect of tungsten on the ALP/2-phospho-l-ascorbic acid trisodium salt reaction was confirmed through the higher affinity of ALP for the substrate in the absence of this metal.

**Figure 2.**
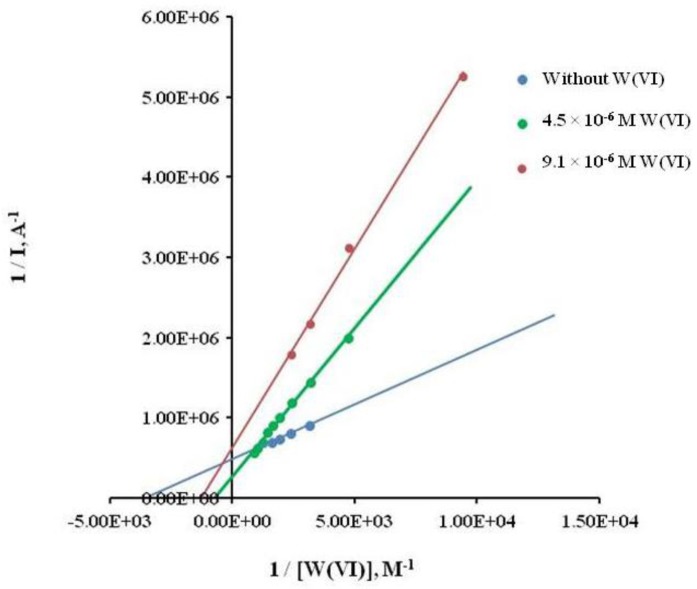
Lineweaver-Burk double reciprocal plot in presence and absence of W(VI) as inhibitor.

As it can be seen from Table 3 and Figure 2, slopes and *K_m_*_apparent_ increases directly with the concentration of W(VI) as inhibitor. In addition, Table 4 shows that *K*_m_ apparent increases directly with ionic strength, so we decided to use an ionic strength of 0.36 M KCl, because the change in current when adding the substrate was higher at this concentration.

### 3.2. Characterization of ALP Biosensor Performance

To characterize an analytical method, it is important to establish its precision in terms of reproducibility and repeatability. The first one was calculated taking into account calibration curves registered using different biosensors (inter-biosensors), and the second one with one single biosensor (intra-biosensor). In this way, several calibration curves were performed in the range of concentration from 0.6 μM to 10 μM at optimum conditions of the experimental variables. Both figures of merit were calculated by the relative standard deviation (RSD) of the slopes from different calibration curves. The reproducibility associated to slopes of these calibration curves in terms of RSD was 4.2% (*n* = 3). Determination of the repeatability was performed similarly using a single ALP based SPCE, which kept 83% of its initial sensibility after the third calibration curve, obtaining a value of 9.4% (*n* = 3) in terms of RSD. Table 5 shows the validated parameters of the calibration curves registered using different biosensors and one biosensor respectively.

The detection of tungsten through the inhibition of ALP/2-PLAs reaction was determined (calibration range from 0.60 μM to 10.0 μM). In this way, the limit of detection based on the standard deviation (3S_y/x_/m) in triplicate of the calibration curve was 0.29 ± 0.01 μM, and the limit of quantification was 0.58 ± 0.02 μM. The performance of the developed procedure was checked by its accuracy and reliability. The accuracy of the proposed method was evaluated by means of the analysis of a tungsten certified sample (High Purity Standards SRM, 1000 ± 4 mg·L^−1^ of W(VI)), and then the biosensor was used to analyse enriched tap water with the same SRM, recovering 991 ± 68% (Table 5). Finally, the developed procedure was applied to the determination of tungsten in spiked tap water samples (1.03 μM), by standard addition methodology with High-Purity Standards traceable to NIST-SRM 3163 by triplicate. The mean concentration of W(VI) was (1.01 ± 0.03) μM (*n* = 3, α = 0.05 and RSD 2.9%, with an average recovery of (97.1 ± 2.9)%. Other samples enriched were purified laboratory water from a TKA System and bottled drinking water, were also enriched with the SRM standard, obtained recoveries of 99.1% ± 2.9% and 99.1% ± 5.2% respectively (*n* = 4).

### 3.3. Interferences

**Figure 3.**
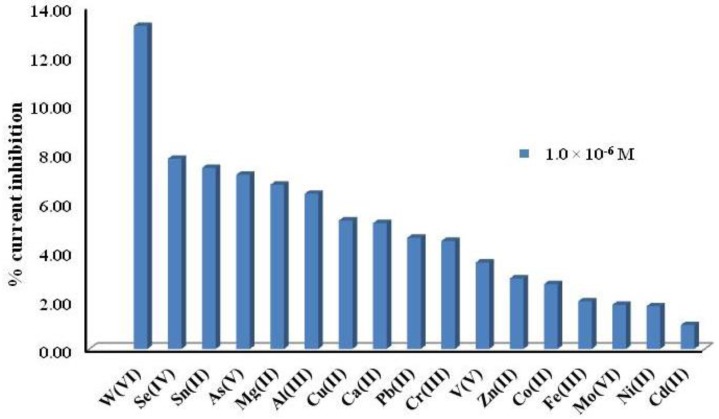
Percentage of inhibition current from several cations at 10^−6^ M, for ALP biosensor Tris HCl buffer pH 8.0, 0.36 M KCl; 0.20 V; 0.32 mM; 2-P-L-Asc as a substrate.

**Figure 4.**
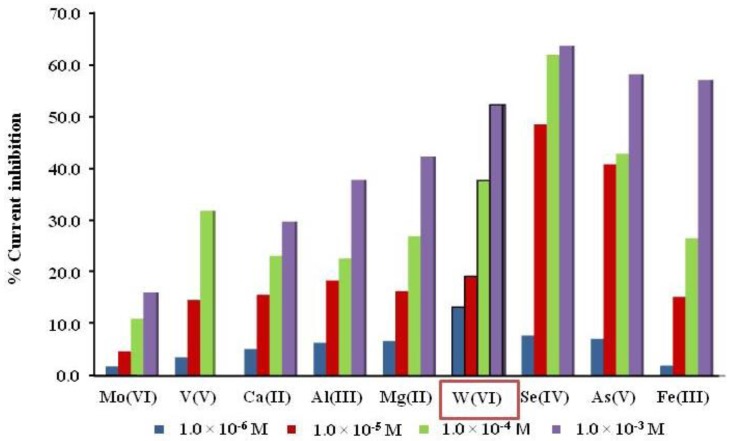
Percentage of inhibition current from several cations at different concentrations, for ALP biosensor, Tris HCl buffer pH 8.0, 0.36 M KCl, 0.20 V, 0.32 mM 2-P-L-Asc as a substrate.

We also studied the effect of different cations at four different concentrations (1.0 μM, 10 μM, 0.1 mM and 1 mM) on the inhibition current of ALP under biosensor-optimized conditions. Their effect was analysed by measuring the inhibition current after consecutive additions of standard solutions of each metal. Figure 3 shows that at μM level, W(VI) produces the highest inhibition current compared to other cations at the same concentration. Nevertheless, Figure 4 presents the inhibition of possible interferences could be present in natural waters such as Ca(II), Al(III), Mg(II) and Fe(III), but also other metals as Se(IV), As(V) and Sn(II). These elements and Fe(III) are major interferences at concentrations higher than 1.0 μM, however if they are present in water at higher concentrations than W(VI), they must be considered in the analysis of tungsten. That is a limiting aspect of this method but we can say that in some cases, many potential interfering cations tend not to exist in many real samples, only Fe(III) should be a problem. In cases where there could be treated with a sample previously precipitant, for example in a basic medium, while other cations are as hydroxides, the W would be as a tungstate. That is to use chemical means of precipitation and complexation to eliminate or decrease the concentration of such interferences. We can say that in view of the good results obtained in spiked real water samples, these interferences does not seem to be a problem.

## 4. Conclusions

The use of ALP based biosensors using AuNPs/SPCEs with 2-PLAsc allows the selective chronoamperometric determination of tungsten. This developed biosensor offers feasibility of use and rapid preparation, low cost and good performance. The figures of merit of the biosensor are adequate to determine tungsten, with a low limit of detection, linear range between 0.6 and 10 μM, and repeatability and reproducibility lower than 10%. There are few interferences at a low concentration of 1.0 μM, nevertheless metals such as Se(IV), As(V), Sn(II), Al(III), and Fe(III) at other concentrations must be taken into consideration in the analysis of tungsten. The effect of tungsten in the ALP/2-PLASc reaction results in a mixed inhibition, which allows the quantitation of tungsten in tap water. The developed procedure shows a limit of detection of 0.29 ± 0.01 μM, and quantitation limit of 0.58 ± 0.02 μM. The reproducibility and repeatability values of RSD for the slopes of several calibrations are lower than 10%. The proposed method can be applicable to different water samples.

## Figures and Tables

**Table 1.** Values corresponding to high (+) and low (–) levels for each factor, used to optimize experimental conditions for tungsten detection.

	**Low Level**	**High Level**	**Optimum**
pH of supporting electrolyte	6.30	9.70	8.00
Substrate concentration	0.036 mM	0.38 mM	0.32 mM
Ionic strength	0.10 M	0.50 M	0.36 M
Working potential	+0.1 V *vs*. Ag/AgCl	+1.0 V *vs*. Ag/AgCl	0.20 V

**Table 2.** Slope variation with applied potential, from different calibration curves for W(VI).

**Applied Potential/V**	**Slope, A/M**
0.20	0.2531
0.30	0.2122
0.40	0.0913
0.50	0.0437

**Table 3.** Michaelis-Menten apparent constant values at different W(VI) concentration.

**W(VI) μg·L^−1^**	**Km**
0	8.14 × 10^−4^
291	1.78 × 10^−3^
740	1.96 × 10^−3^

**Table 4.** Michaelis-Menten apparent constant values at different KCl concentration.

**Conditions**	**Km**
0.10 M KCl without W(VI)	4.32 × 10^−4^
0.10 M KCl with W(VI)	7.49 × 10^−4^
0.25 M KCl with W(VI)	1.67 × 10^−3^
0.50 M KCl with W(VI)	3.54 × 10^−3^

**Table 5.** Precision parameters obtained through ordinary least square (OLS) regression for W(VI) using one or different ALP modified SPCEs under optimum conditions on Table 1.

**Reproducibility**			**Repeatability**	
	
**Electrode**	**Slope A/M**	**R^2^**	**Electrode 3 Slope A/M**	**R^2^**
1	0.0629	0.9974	0.0609	0.9902
2	0.0568	0.9966	0.0537	0.9932
3	0.0609	0.9906	0.0507	0.9930

Media	0.0595		0.0551	
Std. Dev.	0.0025		0.0052	
RSD%	4.2		9.4	
